# Non-target Effects of Hyperthermostable α-Amylase Transgenic *Nicotiana tabacum* in the Laboratory and the Field

**DOI:** 10.3389/fpls.2019.00878

**Published:** 2019-07-09

**Authors:** Ian Melville Scott, Hong Zhu, Katherine Schieck, Amanda Follick, L. Bruce Reynolds, Rima Menassa

**Affiliations:** London Research and Development Centre, Agriculture and Agri-Food Canada, London, ON, Canada

**Keywords:** *Pyrococcus furiosus* α-amylase, *Nicotiana tabacum*, risk assessment, non-target effects, tobacco aphid *Myzus nicotianae*, tobacco hornworm *Manduca sexta*, molecular farming, genetically modified plant

## Abstract

Thermostable α-amylases are important enzymes used in many industrial processes. The expression of recombinant *Pyrococcus furiosus* α-amylase (PFA) in *Nicotiana tabacum* has led to the accumulation of high levels of recombinant protein in transgenic plants. The initial steps to registering the transgenic tobacco at a commercial production scale and growing it in the field requires a risk assessment of potential non-target effects. The objective of this study was to assess the effect of feeding on transgenic tobacco with 2 indigenous insect species commonly associated with wild and commercial tobacco involving plants grown and evaluated under laboratory and field conditions. The highest levels of PFA ranged from 1.3 to 2.7 g/kg leaf fresh weight produced in the field-grown cultivars Con Havana and Little Crittenden, respectively. These two cultivars also had the highest nicotine (ranging from 4.6 to 10.9 mg/g), but there was little to no negative effect for either tobacco hornworm *Manduca sexta* L. or aphid *Myzus nicotianae* (Blackman). Both laboratory and field trials determined no short term (5 days) decrease in the survival or fecundity of the tobacco aphid after feeding on PFA transgenic tobacco compared to non-transgenic plants. In the field, tobacco hornworm larvae showed no differences in survival, final larval weights or development time to adult stage between transgenic lines of four cultivars and their corresponding wild type controls. Laboratory studies confirmed the field trial results indicating the low risk association of PFA expressed in tobacco leaves with tobacco hornworms and aphids that would feed on the transgenic plants.

## Introduction

Evaluating the impact of transgenic or genetically modified (GM) crops to the environment poses unique challenges to the traditional risk assessment process. As more acreage is planted with transgenic crops there will be further interaction of these plants with insects creating new environmental and pest management concerns. The amount of acreage growing transgenic crops increased a 100-fold over the decade between 1996 and 2006, and made up 134 MHa in 25 countries by 2009 (Ahmad et al., 2012), with GM soybeans grown on one of the largest cultivated areas (Imura et al., 2010). A major emphasis of genetic engineering has been to improve crop yield by overcoming abiotic stresses, for example heavy metals, salt, cold and drought. Plants have also been engineered to overcome biotic stresses, to provide defenses against insects, fungi, bacteria and other diseases. The most common insecticidal trait is *Bacillus thuringiensis* (Bt) crystal protein endotoxin that is toxic to lepidopterans (caterpillars), dipterans (flies) and coleopterans (beetles). Other proteins with insecticidal activity are under development, including lectins, protease inhibitors, antibodies, wasp and spider toxins, microbial insecticides and insect peptide hormones (Ahmad et al., 2012). In the United States, stringent rules and regulations have been developed by the National Institutes of Health (NIH), the Department of Agriculture (USDA), the Animal and Plant Health Inspection Service (APHIS), the Environmental Protection Agency (EPA) and the Food and Drug Administration (FDA) that provide guidelines for testing and commercial release of GM crops. The classification of species that may be affected by GM crops include: (1) beneficial, pollinators, and natural enemies; (2) non-target herbivores; (3) soil organisms; (4) species of conservation concern and (5) species of local biodiversity importance (Andow and Zwahlen, 2006). In the European Union, Environmental risk assessment (ERA) of effects on non-target organisms has been adopted for those countries wanting to introduce GM crops for cultivation purposes. Two new guidance documents introduce initiatives that examine ecological principles underlying ERA, such as the requirement of evaluating the whole GM plant, as well as the introduced traits, since the genetic modification may cause unintended changes to the plant’s phenotype that in turn might affect non-target organisms (Arpaia et al., 2017). This is particularly important for new GM crops expressing new traits such as novel proteins as there can be altered plant metabolic pathways (e.g., starch production, oil composition, semiochemicals, etc.) that could cause possible secondary or indirect effects.

The sales from enzymes for biotechnology in the United States increased from $1.3 billion in 2002 to US $5.1 billion in 2009 (Sarrouh, 2012). Fermentation was used traditionally to produce enzymes, but this has been improved with recombinant technology so that microorganisms can produce greater amounts of more active enzymes. Protein engineering can also modify the properties of enzymes (Demain and Vaishnav, 2009). This has led to the successful increased production of thermostable amylase, among other important enzymes (Haki and Rakshit, 2003). To solve the problems associated with recombinant enzymes expressed in *E. coli*, such as inclusion bodies and protein folding issues, yeasts, fungi, plants and animals offer alternative hosts.

Plant expression systems have many positive attributes, but there has been a strong reluctance among regulators to permit agricultural-scale cultivation of transgenic plants expressing foreign proteins (Twyman et al., 2003). Tobacco remains one of the strongest candidates for the commercial production of recombinant proteins, and arguably has the longest history as a successful system for molecular farming (Tremblay et al., 2010). Biosafety and ethical concerns are also satisfied because tobacco is neither a feed nor food crop, eliminating the risk that transgenic tobacco will contaminate animal or human food chains. Of particular interest for this type of study are plant expression systems that produce enzymes, for example amylases, due to the current ability to produce high enzyme concentrations in plants using recombinant technology.

Amylases are important enzymes of great significance in present-day biotechnology. Amylase application includes starch saccharification in the textile, food, brewing, and distilling industries. Since α-amylases need to be active at the high temperatures of gelatinization (100–110°C) and liquefaction (80–90°C) to economize processes, there has been much interest in identifying novel thermostable amylases. The advantages of using thermostable amylases in industrial processes include the decreased cost of external cooling, a better solubility of substrates, and a lower viscosity (de Souza and de Oliveira Magalhaes, 2010). The development of saccharifying amylolytic enzymes such as α-amylase to use with production processes at higher temperatures requires a new process design and improved knowledge of thermophilic microorganisms. One of the most studied thermophilic microorganisms, *Pyrococcus furiosus*, is an anaerobic marine heterotrophic archeon with an optimal growth temperature of 100°C. Alpha-amylase activity has been reported in the cell homogenate and growth medium of *P. furiosus*, and recombinant *P. furiosus* α-amylase (PFA) was expressed and purified from *E. coli* (Dong et al., 1997).

The expression and yield of recombinant PFA in a variety of *Nicotiana* hosts was investigated by transient expression. When plant leaves were infiltrated with *Agrobacterium* carrying codon-optimized PFA DNA sequence, the expression level of PFA was observed to vary among the hosts (Conley et al., 2011). This result suggested that by testing different tobacco cultivars, the accumulation level of recombinant protein could be largely increased. Stable transgenic plants were created with 16 *Nicotiana* cultivars and a total of 438 independent transgenic plants were generated and evaluated (Conley et al., 2011). In Canada, the Canadian Food Inspection Agency (CFIA) regulates the release of transgenic crops. Therefore, two CFIA-approved confined field trials were conducted over two growing seasons to evaluate PFA transgenic plants for crop growth, leaf yield, soluble protein yield, stability of gene expression, the yield of recombinant PFA and alkaloid concentration under field conditions (Zhu et al., 2017). The highest level of PFA measured was 3.4 g/kg fresh weight in field tobacco, which was purified using both chromatography and heating methods. This study is an investigation of the non-target effects of PFA transgenic tobacco that involved both greenhouse and field-grown plants that were evaluated under laboratory and field conditions. Two indigenous insect species commonly associated with wild and commercial tobacco, the tobacco aphid *Myzus nicotianae* (Blackman) (Hemiptera: Aphididae) and the tobacco hornworm *Manduca sexta* L. (Lepidoptera: Sphingidae) were selected to test non-target effects of recombinant PFA on the population of either insect through inhibiting or promoting their growth and development.

## Materials and Methods

### Tobacco Plants

Four cultivars and lines of T1 GM tobacco containing a single insertion of the pfa transgene (Genbank accession number AF001268) were chosen based on the level of α-amylase expression and nicotine content previously determined (Conley et al., 2011) to provide 4 different combinations as follows: high α-amylase, high nicotine (Con Havana 38 line 7F8); low α-amylase, high nicotine (Little Crittenden, line 9F28); high α-amylase, low nicotine (81V9, line 10F18); low α-amylase, low nicotine (TI95, line 3F5). As nicotine is one of the most toxic secondary metabolites in tobacco, it was predicted that the combination of nicotine and the introduced amylase protein might lead to negative effects to herbivores feeding on the transgenic tobacco. Non-transgenic lines of the same cultivars were grown for comparison with the same transgenic lines. The pCaMterX plant expression vector (Harris and Gleddie, 2001) was used for PFA expression. The double enhanced 35S promoter and Nos terminator were the control elements driving gene expression. Tobacco secretory signal peptide Pr1b targeted the protein to the secretory pathway and the KDEL tag retrieved PFA to the ER (Zhu et al., 2017). Activity of PFA was determined by Zhu et al. (2017) by zymography.

### Tobacco Leaf Alkaloid Analyses

Alkaloids were extracted and nicotine quantified using samples from tobacco leaves of each cultivar and line. The leaves were sampled when the plants were green with no senescing leaves. All the leaves were green and healthy (Supplementary Figures S1A–E). Freeze-dried samples were ground in a Wiley mill and the ground material passed through a 2 mm screen and samples were extracted and analyzed by GC-MS using a method previously developed (Kaldis et al., 2013). Briefly, to each 1.0 g sample of ground tobacco, 10 mL of distilled water, 5 mL of dichloromethane (DCM) 5 mL of aqueous 10% sodium hydroxide and 5 mL of DCM containing the internal standard, anethole (Sigma-Aldrich, St. Louis, MO, United States), were added and the mixture shaken for 10 min and centrifuged. An aliquot of the DCM layer was then filtered and analyzed by GC-MS (Hewlett-Packard 5890 Series II GC and 5971A MS detector). A DB-5 capillary column was used (J and W, 60 m × 0.25 mm, 0.25 μm film thickness) (Agilent Technologies, Santa Clara, CA, United States) with the following conditions: 220°C injector temperature; 2.4 mL/min helium flow rate; initial column temperature 50°C for 0.5 min; increased at 5°C/min to 125°C; increased at 2°C/min to 155°C; held for 8 min; increased at 25°C/min to 260°C and held for 8 min. The MS transfer line temperature was 290°C. A selected ion of m/z 162 was used for the nicotine analyses with nicotine standards (Sigma-Aldrich, St. Louis, MO, United States).

### Insects

Tobacco hornworm *M. sexta* were purchased from Carolina Biological (Burlington, NC, United States) as eggs. The eggs were placed on leaves of the tobacco lines that the hornworm larvae would be fed over the course of the laboratory and field trials. Hornworm were kept in an insectary at 25 ± 2°C, 50 ± 5% RH and 16:8 L:D. Tobacco aphid *M. nicotianae* originally collected from field tobacco were maintained as a colony on commercial cultivars of tobacco grown in growth cabinets at 25 ± 2°C, 50 ± 5% RH and 16:8 L:D.

### Laboratory Trials

#### Hornworm Bioassays on Whole Plants

All tobacco cultivars and lines used in the insect lab trials were started from seed, grown in the greenhouse for up to 30 days, and used in the laboratory trials as required. Hornworm larvae hatched from eggs onto leaves from the GM and NGM tobacco cultivars and fed until 2nd instar. Ten 2nd instar hornworm larvae per plant were held on individual leaves using a mesh bag (Delnet^®^ Apertured Film pollination bags, 10″ × 12″) tied closed around the stem with a twist-tie close to the main stalk. Larvae were moved to a fresh leaf on the same plant when they had consumed most of the leaf. When larvae reached the size where they were consuming greater than 1 leaf per day they were moved to a plastic tub (32 × 10 × 17 cm) half to three quarters full of potting soil and fed with fresh tobacco leaves from the same cultivar until they pupated in the soil. The weight of larvae was taken on days 0, 5, 10 and each day prior to pupating. The number of days required to pupate and the date of adult eclosion were recorded. Two replicate trials were performed under growth cabinet conditions of 25 ± 2°C, 50 ± 5% RH and 16:8 L:D.

### Field Trial

The tobacco plants were grown as described by Zhu et al. (2017). The 4 cultivars with both a GM and corresponding NGM line were grown in the field. One and a half to 2 months after transplanting of 6 week-old seedlings, 3 aphid trials of 5 days duration were performed in the field plots. Each trial was set up in a separate block where 4 NGM and 4 GM plants of each cultivar were enclosed by individual wire-framed, mesh-covered cages. Ten < 1 week old aphid adults were held on the top 3rd, 5th, and 7th leaves of each plant using the mesh bags described above. After 5 days, the leaves were cut off the plant at the stalk, and the aphids on each leaf were counted. Plants and aphids were then autoclaved and disposed of in the solid waste.

Two months after planting, a hornworm field trial was set up with 10 2nd instar larvae individually caged per leaf on each plant for 4 NGM and 4 GM plants and enclosed as previously described. After 7 days, the mesh bags with enclosed leaf and larva were removed, and the survival and weight of each were assessed. In the lab, larvae were then placed in individual soil tubs and fed leaf material removed from field plot plants in the same cultivar that they had fed on while in the field. The final larval weight, the number of days as pupae, and the date of adult eclosion were recorded. The hornworm field trials were replicated twice in separate blocks. Plants and hornworms were autoclaved and disposed of in the solid waste.

### Statistics

The nicotine and PFA concentrations from greenhouse-grown and field grown tobacco plants were compared across cultivar and lines using a two-tailed *T*-test (Microsoft Excel 2016).

Survival curves for tobacco hornworm lab and field trials were calculated by the Kaplan-Meier method with comparisons performed based on the log-rank test using IBM SPSS Statistics 20.0 (IBM Corp., United States). ANOVA and unprotected Tukey tests were used to determine if aphid fecundity, and the weight gain and development time of hornworm larvae fed on transgenic (GM) tobacco lines were significantly different from those fed on non-transgenic (NGM) plants (2001; SAS Institute, Cary, NC, United States). Three-way ANOVA and Tukey tests were used to determine if there was an effect of trial in the cases where two trials were used to assess aphid fecundity and hornworm development time and growth on field grown tobacco. If the interaction between trial x line was significant (*P* < 0.05), then the trials were analyzed separately. A two way ANOVA with PROC MIXED was used to determine if there were main effect interactions in the experiments where there was a random plant effect: aphid fecundity over 5 days on field grown tobacco; tobacco hornworm larval day 5 weights on greenhouse grown tobacco; and tobacco hornworm larval day 7 weights on field grown tobacco, as the insects remained on different leaves of the same plant during the feeding period. All other development and growth periods were analyzed by two-way ANOVA with PROC GLM as larvae had been removed from a single plant, held in a soil tub, and were fed leaves from multiple plants of the same cultivar and GM/NGM type until they reached the pre-pupal stage.

## Results

The cultivars and lines selected for the study were based on PFA and alkaloid levels measured in earlier research (Conley et al., 2011). The changes that occur when grown in the field did show a similar trend in alkaloid levels (Table 1) but there were differences in the low and high PFA (Table 2) for the selected lines (Zhu et al., 2017), even though the numbers were not exactly the same as reported in Conley et al. (2011). The PFA levels in the greenhouse grown plants were not measured, however, the transgenic lines were homozygous and reflect relative protein levels as those grown in the field and reported in the Zhu et al. (2017).

**TABLE 1:** The concentration of nicotine (mg/g) in fresh tobacco leaves from the field trial.

		**Concentration (mg/g)**	
**Cultivar**	**Line**	**First harvest**	**Second harvest**
TI 95	3F5	1.10 ± 0.15^B^	0.97 ± 0.13^B^
	NGM	0.97 ± 0.24^b^	0.72 ± 0.23^b*^
C. Havana 38	7F8	7.62 ± 2.13^C^	9.91 ± 1.68^D^
	NGM	8.68 ± 4.45^c^	4.45 ± 1.09^c*^
L. Crittenden	9F28	4.84 ± 0.35^C^	5.12 ± 0.59^C^
	NGM	3.66 ± 1.16^c^	5.39 ± 0.50^c^
81V9	10F18	0.20 ± 0.03^A^	0.30 ± 0.06^A^
	NGM	0.30 ± 0.05^a^	0.27 ± 0.05^a^

The concentration of nicotine is presented as the mean ± SE of four field plot replicates. Mean concentrations of GM lines with the same upper case letter are not statistically different (Two-tailed *T*-test, *P* > 0.05). Mean concentrations of NGM lines with the same lower case letter are not statistically different (Two-tailed *T*-test, *P* > 0.05). An^*^ indicates there was a significant difference between nicotine concentration for 2 NGM lines between first and second harvest (Two-tailed *T*-test, *P* < 0.05).

**TABLE 2:** The concentration of PFA (g/kg fresh tobacco leaf) from the field trial.

		**Concentration (mg/g)**	
**Cultivar**	**Line**	**First harvest**	**Second harvest**
TI 95	3F5	2.47 ± 0.44	0.41 ± 0.13
C. Havana 38	7F8	1.97 ± 0.59	1.24 ± 0.31
L. Crittenden	9F28	2.09 ± 0.62	2.67 ± 0.92
81V9	10F18	0.21 ± 0.04	0.07 ± 0.01

*The concentration of PFA is presented as the mean ± STDEV of measures from 20 individual plants. Data was summarized from Zhu et al. (2017).*

### The Level of Alkaloids in Tobacco

In greenhouse grown tobacco, the alkaloid levels were consistent between the 2 sets of plants: C. Havana and L. Crittenden consistently had higher alkaloid levels than TI95 and 81V9 (Table 3). The same pattern held true for field-grown tobacco and the alkaloids levels are similar between GM and NGM plants within same cultivar (Table 1).

**TABLE 3:** Nicotine concentration (mg/g) of greenhouse-grown tobacco plants.

**Cultivar**	**Line**	**Concentration (mg/g)**
TI 95	3F5	0.55 ± 0.16^A^
C. Havana 38	7F8	3.41 ± 0.29^B^
L. Crittenden	9F28	2.93 ± 0.24^B^
81V9	10F18	0.32 ± 0.10^A^

*The concentration of nicotine is presented as the mean ± SE of measures from five individual plants. Mean concentrations with the same upper case letter are not statistically different (Two-tailed *T*-test, *P* > 0.05).*

### Effect of Recombinant PFA on Aphid Survival and Fecundity

**FIGURE 1:**
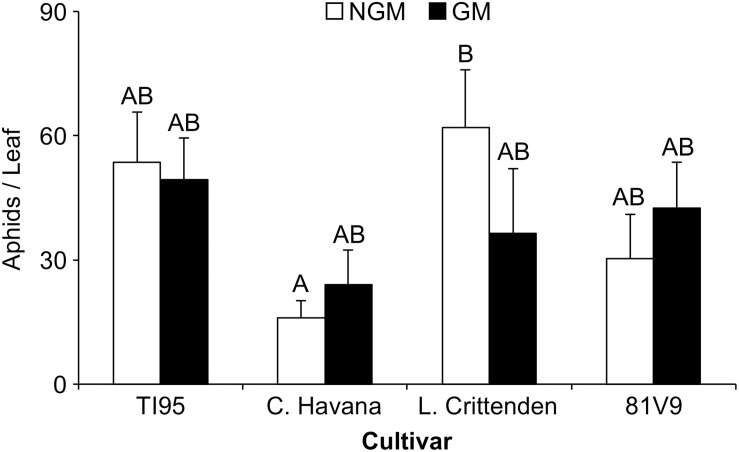
PFA has no effect on short-term tobacco aphid survival and fecundity. Mean aphid numbers ± S.E. on GM and NGM tobacco cultivars. Aphid numbers were averaged between 3 leaves per plant for 3 separate trials under field conditions. Bars with the same letter are not statistically different (2-way ANOVA, Tukey’s pairwise comparison, *P* < 0.05). Error bars represent the standard error of the mean.

To determine if PFA has an effect on aphid survival and fecundity, a trial was conducted where GM and NGM tobacco lines grown in the field were used to rear tobacco aphids. This trial indicated that there was no short term (5 days) effect on the survival or fecundity of the tobacco aphid after feeding on PFA transgenic tobacco compared to the NGM counterpart (Two-way ANOVA: main effects (PROC MIXED) interaction; tobacco line x type; *P* = 0.3346) (Supplementary Table S1), although in one case the NGM C. Havana and had significantly fewer aphids than NGM L. Crittenden (Tukey test, *P* < 0.0001) (Figure 1).

### Effect of Recombinant PFA on Tobacco Hornworm

#### Survival

**FIGURE 2:**
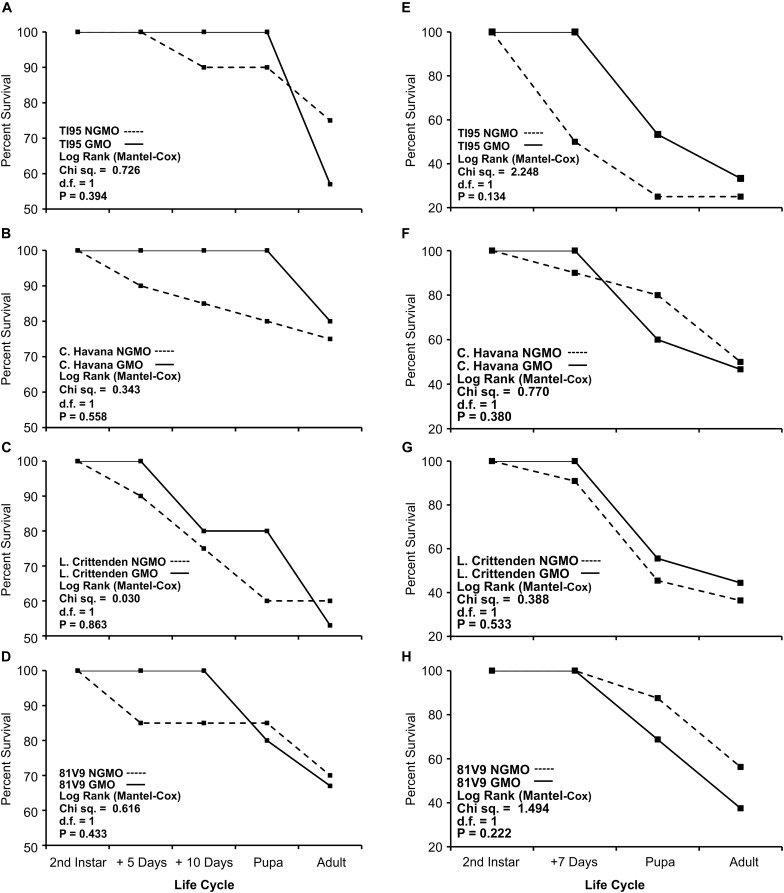
PFA has no effect on tobacco hornworm survival. Survivorship of hornworm from larvae to adult stage on GM vs. NGM tobacco. **(A–D)** Greenhouse conditions and **(E–H)** field conditions (*N* = 20 larvae/cultivar). No significant differences between GM and NGM plants were found (Kaplan-Meier, *P* > 0.05).

To determine if PFA or any other modification introduced during the transformation procedure has any effect on survival of the tobacco hornworm, survivorship assays were conducted on GM and NGM tobacco where the development and survival of hornworm larvae was followed from 2nd instar to the adult stage. There was no significant difference between GM and NGM tobacco (*P* > 0.05) in hornworm survival from larvae reared to the adult stage on greenhouse grown plants (Figures 2A–D).

In the field, 2nd instar hornworm larvae were caged for 7 days on GM and NGM tobacco plants grown in the field and then transferred to the growth chamber and fed leaves cut from the same field grown plants until they reached the pre-pupal stage. Hornworm survival was not significantly different (*P* > 0.05) between the GM and NGM plant lines (Figures 2E–H).

#### Development Time

Other than survival, the length of time a tobacco hornworm larva takes to go through its various life cycle stages can be indicative of a positive or negative effect of PFA.

**FIGURE 3:**
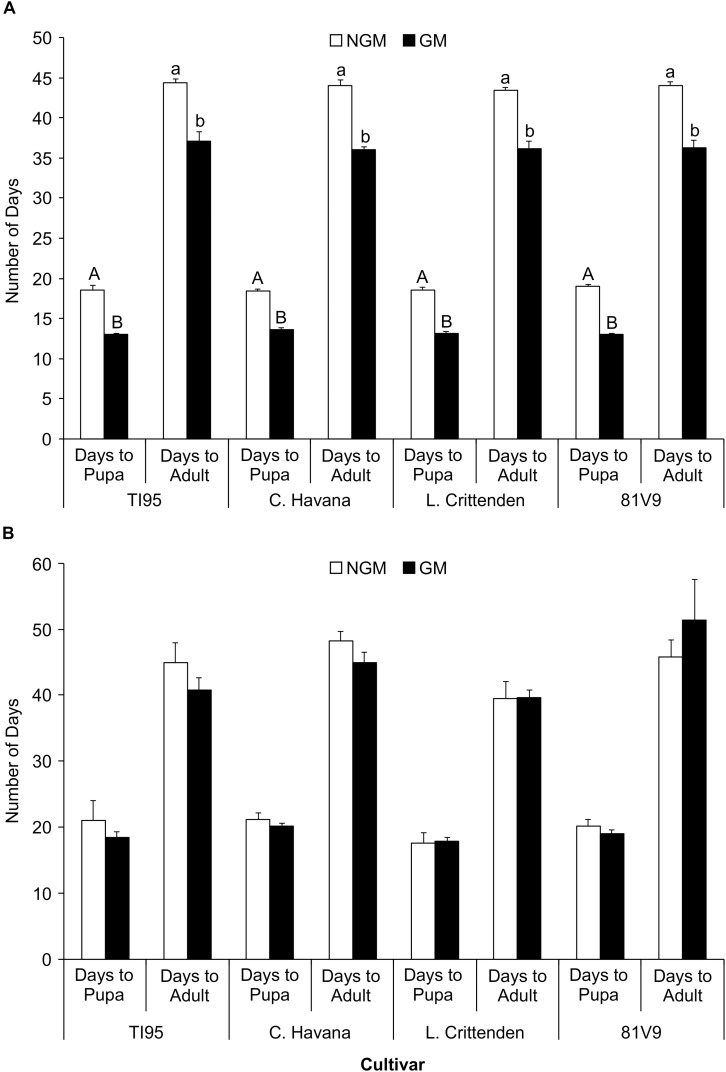
Tobacco hornworm develop faster on transgenic cultivars in the greenhouse, but not in the field. The mean number of days to pupa and to adult stage ± S.E. for tobacco hornworm. **(A)** greenhouse-grown (*N* = 20/cultivar) and **(B)** field-grown plants (*N* = 20/cultivar). Cultivars with the same uppercase (days to pupa) or lowercase (days to adult) letter in **(A)** are not statistically different, no letters in **(B)** indicates no statistical difference among all treatments (2-way ANOVA, Tukey’s pairwise comparison, *P* < 0.05).

The number of days for the larvae on each transgenic cultivar to reach the pre-pupal and adult stage was no different between cultivars (Two-way ANOVA: main effects (PROC GLM) interaction: tobacco line x type; *P* = 0.7417 and *P* = 0.3827, respectively) for the greenhouse-grown tobacco (Supplementary Table S2). However, the development time was significantly less to reach pupal stage and adult stage (Tukey test, *P* < 0.05) for larvae fed on GM than on the NGM lines of the same cultivar (Figure 3A). The GM and NGM plants were grown and tested under identical environmental conditions but at different times (3 months apart) which may have contributed to the observed differences. Analysis of covariance with time as the covariant could not be tested in this case as the treatments were different during each trial (NGM lines in first trial and GM lines in the second). A two way ANOVA was applied based on two types of plants (GM vs. NGM) and 4 cultivars. In contrast, there was no difference in the length of time for the larvae to reach the pre-pupal stage or the time until adult emergence (Two-way ANOVA: main effects (PROC GLM) interaction: tobacco line x type; *P* = 0.7417 and *P* = 0.3827, respectively) (Supplementary Table S3) for all larvae on field-grown tobacco cultivars in both trials (Figure 3B). As the GM and NGM plants were tested at the same time under field conditions, these results are more illustrative of the lack of effect GM tobacco cultivars have on hornworm development time. These results also reinforce the importance of using appropriate controls and conducting all experiments simultaneously, as the varying physiological conditions of the plants and/or the insects may influence experimental outcomes.

#### Growth

A third factor that can inform us on any effects of PFA is weight gain of hornworm larvae while feeding on GM and NGM plants. To determine whether growth on the GM lines with both higher nicotine and PFA would affect survival to the pupal and adult stages, the hornworm larvae were held on the plants over the entire larval period.

**FIGURE 4:**
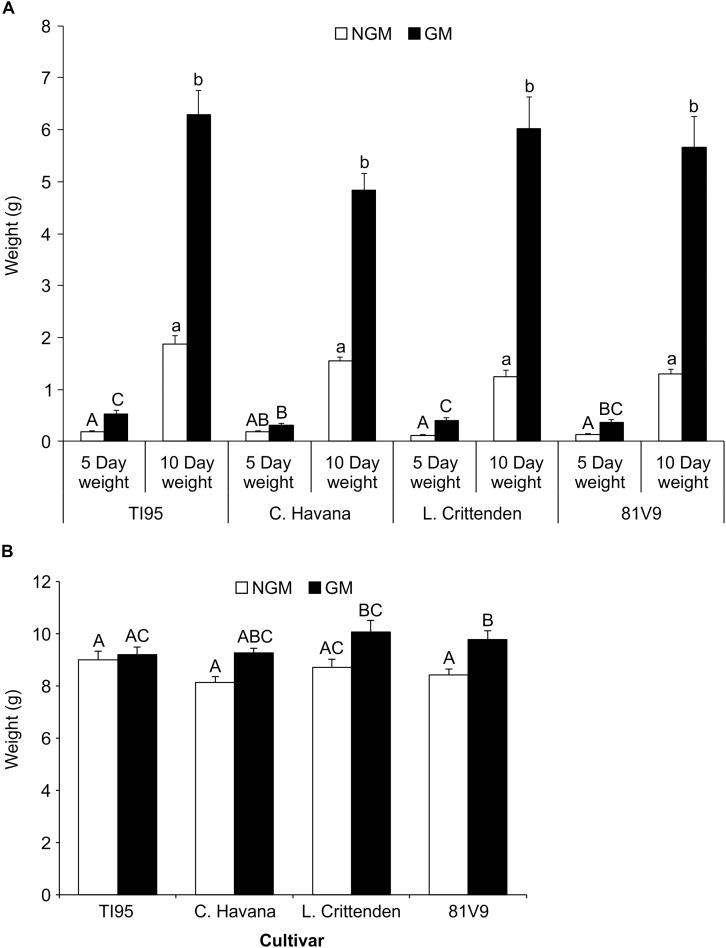
Tobacco hornworm larvae gain more weight on greenhouse-grown transgenic lines when younger, but even-out by the pre-pupal stage. Mean larval weights ± S.E. of tobacco hornworm larvae fed on greenhouse grown, non-transgenic and transgenic tobacco cultivars (*N* = 20 larvae/cultivar). **(A)** 5 and 10 days old larvae and **(B)** pre-pupal larvae (after approximately 20 days). Bars with the same uppercase (5 days weights) or lowercase (10 days weights) letter in **(A)** are not statistically different, while bars with the same uppercase letter in **(B)** indicates no statistical difference (2-way ANOVA, Tukey’s pairwise comparison, *P* < 0.05).

The 2nd instar larvae fed on greenhouse plants until the pre-pupal stage, there was no significant main effect interaction at Day 5 for larvae fed on GM compared to NGM cultivars (Two-way ANOVA: main effect (PROC MIXED) interaction; tobacco line x type; *P* = 0.1297) (Supplementary Table S4), however, GM TI95, L. Crittenden and 81V9 had significantly greater larval weight than those fed on the corresponding NGM plants (Tukey test, *P* < 0.05) (Figure 4A). Similarly on Day 10, there was no significant main effect interaction for larvae fed on GM compared to NGM plants (Two-way ANOVA: main effect (PROC GLM) interaction; tobacco line x type; *P* = 0.2113) for each cultivar (Figure 4A). However, all larvae fed on GM plants had significantly greater weights than those fed on the corresponding NGM plants (Tukey test, *P* < 0.05). By the pre-pupal stage, larvae feeding on NGM plants had caught-up in weight, and only hornworms that fed on GM 81V9 had a significantly greater (Two-way ANOVA: main effect (PROC GLM) interaction; tobacco line x type; *P* = 0.1397) final weight than those on the NGM 81V9 (Tukey test; *P* < 0.05) (Figure 4B). These results indicate that PFA has a somewhat positive effect on weight gain (Figure 4A) and development time (Figure 3A) of hornworm larvae fed greenhouse grown tobacco. This could be due to conversion of starch to sugar, making the plants more palatable to larvae, but this effect seems to be transient, since by the time they stop feeding, most larvae are in the same weight range whether fed GM or NGM plants.

**FIGURE 5:**
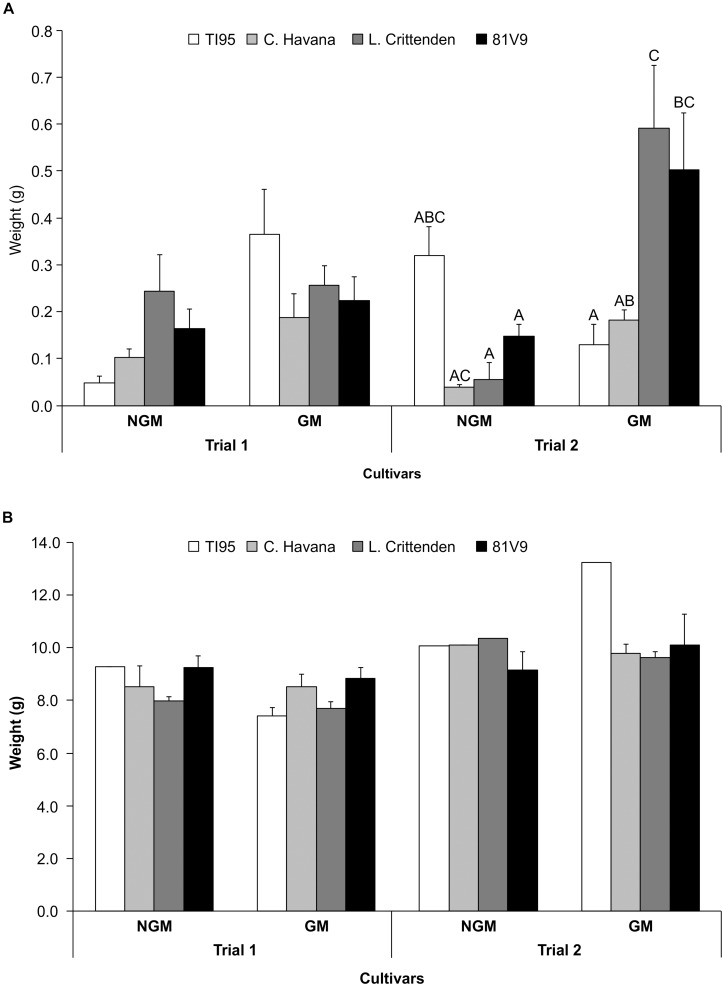
PFA has no effect on final tobacco hornworm weight in field conditions. Mean larval weight ± S.E. on field-grown tobacco (*N* = 20 larvae/cultivar) in two field trials. **(A)** 7 days hornworm larval weight and **(B)** pre-pupal larval weight (after approximately 20 days). Bars with the same letter in **(A)** trial 2 are not statistically different, no letters in **(A)** trial 1 and in **(B)** trials 1 and 2 indicates no statistical difference among all treatments (2-way ANOVA, Tukey’s pairwise comparison, *P* > 0.05).

In the field, there was a significant main effect interaction of trial x line (*P* = 0.0403) (Supplementary Table S5), therefore the effect of the tobacco line and type were analyzed separately for the 2 trials. There was no significant larval weight differences observed at 7 days between GM and corresponding NGM cultivars in Trial 1 (Two-way ANOVA: main effect (PROC MIXED) interaction; tobacco line x type; *P* = 0.2260) (Figure 5A), but in Trial 2 there were weight differences between GM and NGM cultivars (Two-way ANOVA: main effect (PROC MIXED) interaction; tobacco line x type; *P* = 0.0441), L. Crittenden and 81V9 (Tukey test, *P* < 0.05). Larvae fed field plants from Day 7 until they reached the pre-pupal stage under growth chamber conditions showed no differences in final larval weights among the same GM and NGM cultivars in both Trial 1 and 2 (Two-way ANOVA: main effects (PROC GLM) interaction: line x type; *P* = 0.6805 and *P* = 0.4650, respectively) (Figure 5B).

## Discussion

In general, the combination of high nicotine and high PFA were not found to increase development time or reduce survival for hornworm fed *in situ* on field leaves for 7 days and then field grown leaves until the larvae reached pre-pupal and adult stages. Significant differences between larval weights during the hornworm development were observed at the mid-larval stage, but under both laboratory and field conditions these differences were no longer observed by the time the larvae reached the pre-pupal stage.

Neither insect species was negatively affected by 4 different PFA transgenic lines, even though differences were observed at times during certain points in the hornworm life-stages. Laboratory studies confirmed what was seen in the field and indicate that short-term bioassays provided appropriate predictions of the response of tobacco hornworms and aphids to recombinant proteins. Based on the current levels of PFA produced in the higher nicotine cultivars, little negative impact should be expected within 1 generation of tobacco hornworm or aphid exposed to GMO tobacco production. Since nicotine and alkaloid levels can vary with tobacco leaf age (Zhang et al., 2018), insects in this study would be exposed to different levels depending on where they feed on the plant. For this reason 3 or more leaves on each plant were selected to position the aphids and in the case of the hornworms, 10 leaves per plant were used in order not to bias the exposure. In addition, all tobacco plants sampled within each trial were the same age, and leaves were removed, stacked on top of each other, and a core taken that would include a sample from all leaves combined. Duplicate samples were obtained from each plant for the alkaloid analyses.

The selection of non-target arthropods (NTAs) is critical to be able to test the hypothesis that GE crops, arthropod-active or not, do not cause adverse effects to valued NTAs under field exposures (Romeis et al., 2013). In this study the arthropods meet the selection criteria since tobacco hornworm and aphids are most likely to ingest the PFA based on their biology, mode of feeding and will be exposed to high levels of the protein for an extended duration.

Tobacco hornworm can consume tobacco leaves in their entirety thus ensuring that any produced transgenic proteins would be ingested during feeding and processed within the insect’s digestive tract. Any adverse effects from α-amylase recombinant protein ingestion could manifest themselves in the form of decreased survival, reduced larval weights or increased development times to pupal and adult stages, but this was not observed with any of the transgenic tobacco tested. Fecundity of tobacco hornworm could also be affected but would require a long term, multi-generational study to determine if such effects were present. Fecundity of the tobacco aphid was assessed on the transgenic and non-transgenic tobacco based on the number of aphid nymphs produced, and no differences were observed. However, aphids feed by piercing the leaf epidermis to access the phloem, and the amount of protein present relative to the amount sequestered in cells is not known. Longer term, multi-generational studies could also be done with this species to determine if there are any chronic effects of lower PFA exposure.

Concerns were addressed over the limitations of sample size used in this study by pointing out that hundreds of tobacco aphids and hornworms were exposed to the transgenic plants in the course of the laboratory and field trials. The initial aphid numbers placed on plants were 10 per leaf, but as aphids reproduce quickly, the populations on all leaves and plants increased by 2- to 3-fold within 5 to 7 days. Overall, 720 aphids were tested under field conditions. This breaks down to at least 30 aphids/plant and 3 plants/treatment. Fewer tobacco hornworm could be tested as growing larvae consumed large amounts of leaf tissue and would require more tobacco plants than time and resources would allow for greenhouse trials. Twenty to thirty larvae were tested per greenhouse grown tobacco cultivar for a total of 160. The numbers of larvae tested under field conditions was also 160.

A great deal of the literature on the non-target effects of transgenic plants focuses on B.t. insecticidal proteins in various crops (Han et al., 2016) and those that have examined the impact of herbicide-tolerant crops on arthropods (Imura et al., 2010). In the latter case, the impact of GM plants was assessed from the perspective of how the plants and their new cropping practices affect the ecosystem of the field versus that of conventional cropping system. A 2 years field study determined that the GM plant itself was not responsible for changes in the arthropods of the soybean fields, but the modified weed management strategies were likely responsible by reducing the types of arthropods present. In the present study, little attention was paid to the diversity of arthropods (insects and others) found on the wild-type versus PFA producing tobacco. Only a few species were collected from within the field plots during the study, and would not provide a significant analysis of non-target effects or impacts to biodiversity. Typically, the tobacco crop would have been treated with insecticide sprays in order to manage tobacco pests such as the hornworm and aphid, but this was not done in order to complete the feeding trials. Based on these results it can be estimated that the recombinant protein does not pose a risk to the insects, but neither are the transgenic plants more susceptible to the insects. Since the amount of leaf area consumed was not calculated, the only evidence was the level of insect growth and time to complete larval development, and these were not significantly different by the end of the larval stage. However, the significantly higher growth during the mid-lifecycle of the larvae fed on the PFA plants may indicate that greater amounts of leaf material are consumed relative to the wild-type plants. A PFA producing plant may be a more nutritious food source for the hornworms, leading to faster growth of the insects, and greater feeding damage on the PFA plants. As was mentioned previously, the conversion of higher levels of starch in PFA plants would provide greater amounts of sucrose, a compound consistently shown to be one of the most stimulating of sugars to insects (Hervé et al., 2014).

The evaluation of non-target effects from transgenic plants often employs indicator species, but this can also include non-target species that are common within the local area where the crops are grown (Andow and Zwahlen, 2006). A more judicious selection process for choosing an indicator or non-target species to assess GM plant risks would include a survey of published literature on the attributes of each non-target invertebrate and the transgenic plant of interest (Todd et al., 2008). The most pertinent outcome of the screening process is the increased confidence in the risk analysis, knowing that all species in the ecosystem have been considered. Such a screening method might include assessment of effects on beneficial insects that do not feed directly on the GM plant, but would feed on herbivores that do consume the plant (Kalushkov and Nedved, 2005). The use of meta-analyses to measure direct toxic effects on non-target organisms is also recommended, as long as the studies are designed to separate direct and indirect effects (Schmidt et al., 2009). Unfortunately, meta-analysis for transgenic PFA- producing tobacco is not possible since this was a preliminary evaluation of this protein in plants. Therefore, evidence can only come from lab and field studies as were conducted in the present study. For example, an investigation of 4 transgenic potato lines that produce cyanophycin, a polymer of aspartic acid and arginine, between 0.8 and 7.5% dry weight in the tubers were compared to a non-transgenic potato variety (Emmerling et al., 2012). Similar to the present study, the residues containing cyanophycin had no effect on subtle behavior, growth or reproduction of earthworms based on endpoints such as loss of potato residue at the soil surface, earthworm biomass, cocoon production and earthworm hatching numbers over an 80 days period. The majority of studies have similarly indicated that non-target species were not affected by transgenic crops; however, the consensus is that each GM plant should be assessed separately based on the gene expressed by the plant.

An opposing view is that the risks associated with the development of transgenic crops expressing recombinant proteins may be assessed with sufficient confidence without most of the ecotoxicological studies required for pesticidal transgenic crops. In fact many of the risks could be evaluated by using information from the scientific literature on the mode of action, taxonomic distribution and environmental fate of the recombinant proteins (Raybould et al., 2010). However, there is potential concern regarding any pleiotropic effects from recombinant traits, for example with the un-intended effects of protease inhibitor gene-expressing crops (Schluter et al., 2010). Un-intended effects within the modified plant would negate the sole use of previous scientific/toxicity data approach and suggest that an empirical assessment on a case-by-case basis is still required.

## Conclusion

Thus far, at the current level of the PFA produced by tobacco, the field trials indicate a low risk to non-target insects. However, future projects should assess the impact of these proteins to the third trophic level, or to predators and parasitoids of the insects that feed on tobacco. Those organisms may not have the same ability to metabolize or detoxify exogenous proteins that are encountered when preying on the herbivores that ingest the proteins.

## Informed Consent

IS has provided his informed consent for the identifiable image shown in Supplementary Figure S1C.

## Author Contributions

IS, LR, and RM designed the research. IS, HZ, KS, and AF conducted the research. IS, HZ, and KS analyzed the data. IS, HZ, and RM wrote the manuscript with contributions from all authors. All authors read and approved the final version of the manuscript.

## Conflict of Interest Statement

The authors declare that the research was conducted in the absence of any commercial or financial relationships that could be construed as a potential conflict of interest.
