# Supplementary material for: Non-target Effects of Hyperthermostable α-Amylase Transgenic Nicotiana tabacum in the Laboratory and the Field
Source: Front Plant Sci. 2019 Jul 9;10:878. doi: 10.3389/fpls.2019.00878 (PMC6630089; doi:10.3389/fpls.2019.00878)
Supplement: Supplementary file 1 [file Image_1.pdf]

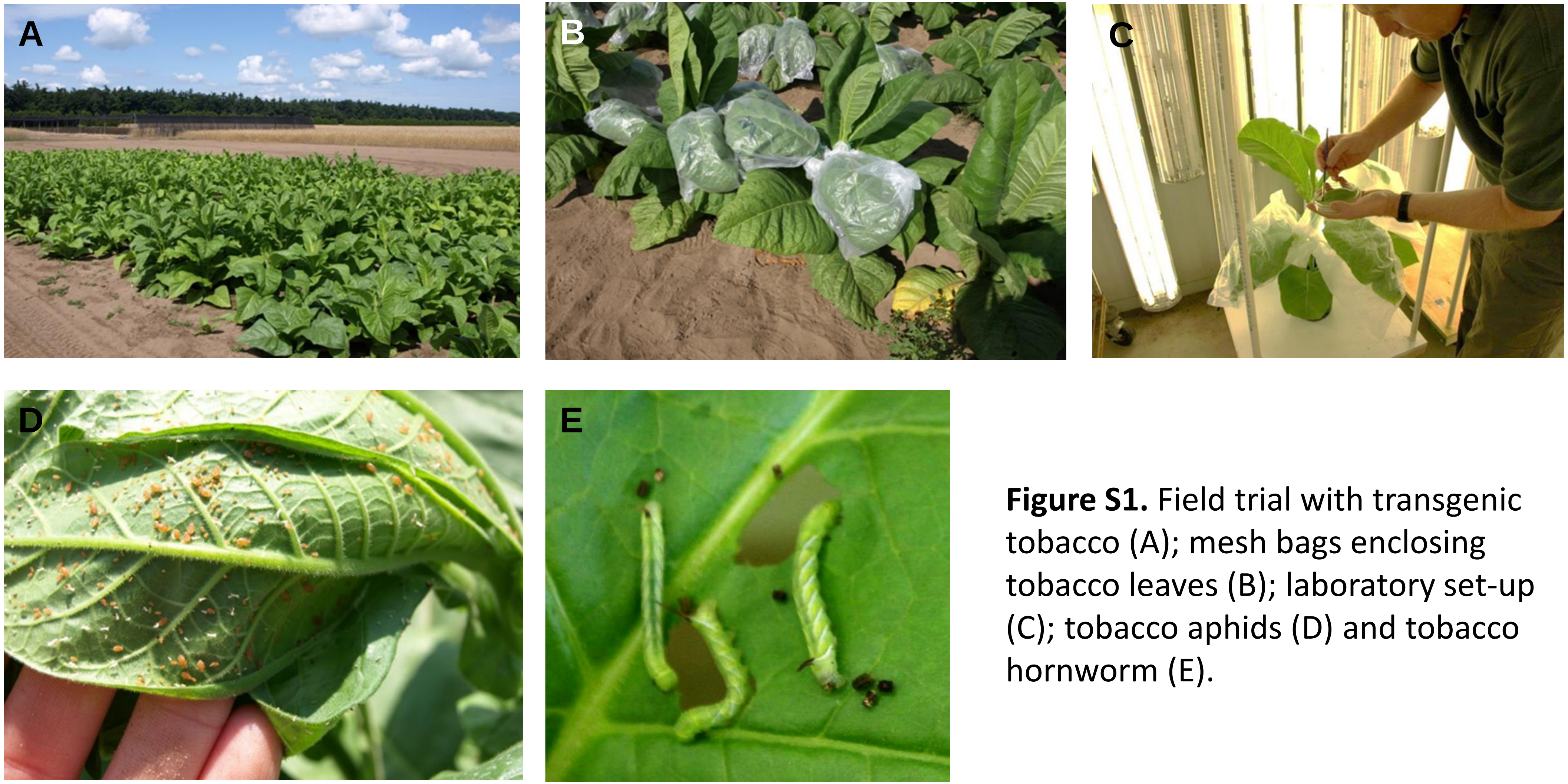

Figure S1. Field trial with transgenic tobacco (A); mesh bags enclosing tobacco leaves (B); laboratory set-up (C); tobacco aphids (D) and tobacco hornworm (E).
