# Supplementary material for: Non-target Effects of Hyperthermostable α-Amylase Transgenic Nicotiana tabacum in the Laboratory and the Field
Source: Front Plant Sci. 2019 Jul 9;10:878. doi: 10.3389/fpls.2019.00878 (PMC6630089; doi:10.3389/fpls.2019.00878)
Supplement: Supplementary file 2 [file Table_1.docx]

Table S1. Tobacco aphid fecundity during 5 days of exposure to transgenic (GM) and non-transgenic (NGM) tobacco lines in the field and two-way analysis of variance.

| Trial | Leaf # | TI95 | | C. Havana | | L. Crittenden | | 81V9 | |
| --- | --- | --- | --- | --- | --- | --- | --- | --- | --- |
|  |  | NGM | GM | NGM | GM | NGM | GM | NGM | GM |
|  |  | Number of aphid nymphs per leaf after 5 days | | | | | | | |
| 1 | 3 | 54 | 74 | 42 | 53 | 113 | 8 | 59 | 26 |
|  | 5 | 101 | 99 | 29 | 5 | 126 | 17 | 77 | 41 |
|  | 7 | 100 | 76 | 14 | 60 | 49 | 148 | 21 | 88 |
| 2 | 3 | 11 | 42 | 12 | 46 | 84 | 39 | 1 | 96 |
|  | 5 | 18 | 45 | 6 | 41 | 76 | 71 | 2 | 67 |
|  | 7 | 56 | 39 | 8 | 4 | 61 | 23 | 0 | 7 |
| 3 | 3 | 27 | 51 | 11 | 2 | 18 | 3 | 78 | 16 |
|  | 5 | 27 | 13 | 10 | 5 | 12 | 15 | 17 | 21 |
|  | 7 | 81 | 6 | 12 | 0 | 19 | 4 | 17 | 21 |
|  | Avg (s.e.) | 53.7  (11.9) | 49.4  (9.98) | 16.0  (3.92) | 24.0  (8.41) | 62.0  (13.9) | 36.4  (15.7) | 30.2  (10.7) | 42.6  (11.0) |

Main effects: GM//NGM type x 2; Tobacco lines x 4; Trials/line/type x 3; Aphids/plant = 30; Total aphids = 720.

Three-way ANOVA - main effects (PROC GLM): line (P=0.0043); type (P=0.7194); trial (P<0.0001). Interactions of main effects: trial x line (P=0.2141); trial x type (P=0.1511); line x type (P=0.1810).

Two-way ANOVA for combined trials - main effects (PROC MIXED): line (P=0.0241); type (P=0.7661). Interactions of main effects: line x type (P=0.3346).
