# Supplementary material for: Non-target Effects of Hyperthermostable α-Amylase Transgenic Nicotiana tabacum in the Laboratory and the Field
Source: Front Plant Sci. 2019 Jul 9;10:878. doi: 10.3389/fpls.2019.00878 (PMC6630089; doi:10.3389/fpls.2019.00878)
Supplement: Supplementary file 3 [file Table_2.DOCX]

Table S2. Tobacco hornworm development time for larvae to pupae and larvae to adult on transgenic and non-transgenic tobacco lines in the laboratory and two-way analysis of variance.

| Life-stage | Plant # | TI95 | | C. Havana | | L. Crittenden | | 81V9 | |
| --- | --- | --- | --- | --- | --- | --- | --- | --- | --- |
|  |  | NGM | GM | NGM | GM | NGM | GM | NGM | GM |
|  |  | Number of days from larvae to each life-stage | | | | | | | |
| Pupa | 1 | 19, 17, 18, 18, 20, 28, 17, 16 | 13, 14, 13, 13, 13, 13, 13, 13, 13 | 18, 17, 19, 18, 17, 19, 19, 19 | 15, 14, 13, 13, 13, 13 | 19, 18, 18, 20, 19, 18, 16 | 13, 13, 14, 14, 13, 13, 15, 13 | 20, 19, 17, 18, 20, 18, 18, 19 | 13, 13, 13, 13, 13, 13, 13, 13, 13 |
|  | 2 | 16, 17, 23, 18, 18, 18, 16, 19, 18, 17 | 13, 12, 13, 12, 13, 13, 14 | 19, 19, 17, 18, 19, 29, 18 | 15, 13, 12, 13, 13, 15, 14, 14, 13, 15 | 20, 18, 19, 18, 20 | 14, 13, 12, 12, 12 | 21, 20, 19, 20, 18, 19, 18, 21 | 14, 13, 13, 13, 13 |
|  | Avg (s.e.) | 18.5  (0.68) | 13.0  (0.13) | 18.4  (0.22) | 13.6  (0.24) | 18.6  (0.34) | 13.1  (0.25) | 19.0  (0.28) | 13.1  (0.07) |
| Adult | 1 | 44, 47, 41, 46, 46, 45, 44 | 38, 34, 35 | 48, 45, 42, 47, 42, 42, 44, 44 | 37, 38, 37, 34 | 44, 42, 43, 42, 42, 44, 45 | 38, 35, 40 | 45, 44, 44, 44, 44, 41, 45 | 35, 35, 39, 37, 34, 43 |
|  | 2 | 41, 46, 44, 45, 44, 45, 44, 44 | 36, 35, 37, 36, 36 | 45, 46, 40, 45, 41, 47, 43 | 36, 36, 34, 34, 37, 36, 36, 37 | 45, 44, 42, 42, 46 | 37, 37, 33, 33 | 46, 46, 45, 47, 41, 42 | 35, 36, 35, 34 |
|  | Avg (s.e.) | 44.4  (0.43) | 37.1  (1.20) | 44.1  (0.61) | 36.0  (0.39) | 43.4  (0.42) | 36.1  (0.99) | 44.0  (0.50) | 36.3  (0.88) |

Main effects: GM//NGM type x 2; Tobacco lines x 4; Plants/line/type x 2; Hornworms/plant = 10; Total hornworms = 160.

Two-way ANOVA - Days to pupa main effects (PROC GLM): line (P=0.7894); type (P<0.0001). Interactions of main effects: type x line (P=0.4624).

Two-way ANOVA - Days to adult main effects (PROC GLM): line (P=0.5228); type (P <0.0001). Interaction of main effects: type x line (P=0.9106).
