# Supplementary material for: Non-target Effects of Hyperthermostable α-Amylase Transgenic Nicotiana tabacum in the Laboratory and the Field
Source: Front Plant Sci. 2019 Jul 9;10:878. doi: 10.3389/fpls.2019.00878 (PMC6630089; doi:10.3389/fpls.2019.00878)
Supplement: Supplementary file 4 [file Table_3.DOCX]

Table S3. Tobacco hornworm development time for larvae to pupae and larvae to adult on transgenic and non-transgenic tobacco lines in the field and two-way analysis of variance.

| Life-stage | Plant # | TI95 | | C. Havana | | L. Crittenden | | 81V9 | |
| --- | --- | --- | --- | --- | --- | --- | --- | --- | --- |
|  |  | NGM | GM | NGM | GM | NGM | GM | NGM | GM |
|  |  | Number of days from larvae to each life-stage | | | | | | | |
| Pupa | 1 | 24 | 14, 17, 19, 17, 19, 22 | 17, 20, 22, 19, 20, 24, 24 | 19, 20, 19, 19, 23, 20 | 15, 19, 16, 15 | 16, 17, 19, 16, 16, 19, 20 | 27, 16, 17, 16, 19, 21, 23, 19, 21 | 19, 19, 22, 22, 16, 17, 19, 19 |
|  | 2 | 18 | 21 | 24 | 21, 20 | 23 | 20 | 20, 17, 27, 20 | 17, 18, 21 |
|  | Avg  (s.e.) | 21.0  (3.0) | 18.6  (0.89) | 21.25  (0.94) | 20.1  (0.48) | 17.6  (1.54) | 17.9  (0.64) | 20.2  (1.01) | 19.0  (0.6) |
| Adult | 1 | 48 | 39, 39, 39, 39 | 48, 50, 43, 49 | 39, 48, 41, 48, 49 | 37, 37,37 | 39, 37, 37, 37, 43, 37, 43 | 40, 40, 40, 64, 43, 48, 48 | 80, 48, 56, 39 |
|  | 2 | 42 | 48 | 51 | 47, 43 | 47 | 44 | 46, 43 | 42, 43 |
|  | Avg (s.e.) | 45.0  (3.0) | 40.8  (1.8) | 48.2  (1.39) | 45.0  (1.5) | 39.5  (2.5) | 39.6  (1.12) | 45.8  (2.52) | 51.3  (6.23) |

Main effects: GM//NGM type x 2; Tobacco lines x 4; Plants/line/type x 2; Trials/line/type x 2; Hornworms/plant = 10; Total hornworms = 160.

Three-way ANOVA - Days to pupa main effects (PROC GLM): line (P=0.0638); type (P=0.0925); trial (P=0.0931). Interactions of main effects: trial x line (P=0.3988); trial x type (0.5391); line x type (P=0.6704).

Two-way ANOVA for combined trials - Days to pupa main effects (PROC GLM): line (P=0.0596); type (P=0.0776). Interactions of main effects: line x type (P=0.7417).

Three-way ANOVA - Days to adult main effects (PROC GLM): line (P=0.0262); type (P=0.6319); trial (P=0.7394). Interactions of main effects: trial x line (P=0.2145); trial x type (0.5916); line x type (P=0.3425).

Two-way ANOVA for combined trials - Days to adult main effects (PROC GLM): line (P=0.0245); type (P=0.6277). Interactions of main effects: line x type (P=0.3827).
