# Supplementary material for: Non-target Effects of Hyperthermostable α-Amylase Transgenic Nicotiana tabacum in the Laboratory and the Field
Source: Front Plant Sci. 2019 Jul 9;10:878. doi: 10.3389/fpls.2019.00878 (PMC6630089; doi:10.3389/fpls.2019.00878)
Supplement: Supplementary file 5 [file Table_4.DOCX]

Table S4. Tobacco hornworm 5 day, 10 day larval and pre-pupal weight on transgenic and non-transgenic tobacco lines in the laboratory and two-way analysis of variance.

| Weighing date | Plant # | TI95 | | C. Havana | | L. Crittenden | | 81V9 | |
| --- | --- | --- | --- | --- | --- | --- | --- | --- | --- |
|  |  | NGM | GM | NGM | GM | NGM | GM | NGM | GM |
|  |  | Number of days from larvae to each life-stage | | | | | | | |
| Day 5 | 1 | 0.13, 0.14, 0.22, 0.14, 0.04,  0.23,  0.05,  0.25,  0.26, 0.26 | 0.71, 0.24, 0.43, 1.48, 0.28, 0.77, 0.72, 0.76, 0.22, 0.32 | 0.25, 0.25, 0.25, 0.27, 0.28, 0.23, 0.25, 0.13, 0.19 | 0.71, 0.21, 0.27, 0.26, 0.20, 0.58, 0.18, 0.57, 0.33, 0.15 | 0.09, 0.25, 0.27, 0.06, 0.14, 0.03, 0.22, 0.05 | 0.28, 0.32, 0.73, 0.28, 0.21,  0.95, 0.49, 0.48, 0.1, 0.70 | 0.23, 0.23, 0.25, 0.21, 0.24, 0.14, 0.21 | 0.39, 0.54, 0.24, 0.61, 0.62, 0.65, 1.02, 0.57, 0.24, 0.24 |
|  | 2 | 0.22, 0.24, 0.26, 0.27, 0.25, 0.26,  0.25,  0.27,  0.28 | 0.39, 0.67, 0.29, 0.69, 0.62, 0.24, 0.50, 0.57, 0.29, 0.25 | 0.05, 0.17, 0.1, 0.1, 0.27,  0.20, 0.15, 0.25, 0.17 | 0.26, 0.27, 0.52, 0.3, 0.49, 0.28, 0.23, 0.24, 0.27, 0.23 | 0.27, 0.04, 0.05, 0.09, 0.14, 0.11, 0.05, 0.02 | 0.07, 0.28, 0.29, 0.59, 0.35, 0.42, 0.64, 0.56, 0.49, 0.01 | 0.06, 0.07, 0.09, 0.11, 0.06, 0.25, 0.01, 0.12 | 0.29, 0.26, 0.21, 0.23, 0.28, 0.25, 0.14, 0.21, 0.27, 0.12 |
|  | Avg (s.e.) | 0.21  (0.02) | 0.52  (0.07) | 0.2  (0.02) | 0.33  (0.03) | 0.12  (0.02) | 0.41  (0.05) | 0.15  (0.02) | 0.37  (0.05) |
| Day 10 | 1 | 1.11, 1.57, 1.48, 1.75, 0.73, 0.40, 2.17, 1.47 | 5.57, 3.5, 7.19, 8.51, 7.39, 9.95, 8.47, 7.93, 4.61, 6.91 | 1.73, 1.77, 1.47, 1.44, 1.77, 1.51, 1.39, 1.56, 1.56 | 3.42, 3.91, 4.01, 6.82, 5.99, 6.24, 4.69, 8.05, 5.79, 4.55 | 1.37, 1.71, 1.93, 0.90, 1.72, 1.53, 0.5, 1.41, 0.83 | 6.68, 6.03, 9.16, 3.7, 1.54, 8.63, 8.73, 6.14, 1.64, 6.5 | 1.34, 1.63, 1.71, 1.72, 1.52, 1.24, 1.41, 1.58 | 5.87, 8.73, 6.3, 5.48, 7.13, 11.0, 8.72, 8.84, 6.76 |
|  | 2 | 2.74, 1.89, 2.35, 1.75, 1.94, 2.41, 3.33, 2.43, 2.12, 2.41 | 5.28, 4.64, 4.2, 7.78, 6.01, 5.74, 3.28 | 0.98, 1.3, 2.14, 1.82, 1.44, 1.90, 1.45, 1.32 | 1.94, 4.91, 6.84, 5.44, 4.81, 2.67, 3.91, 4.22, 4.99, 3.58 | 0.71, 0.88, 1.33, 1.63, 1.63, 0.83 | 5.18, 4.69, 8.41, 6.83, 6.57 | 0.80, 0.98, 1.07, 1.38, 1.17, 1.52, 0.24, 1.32 | 2.84, 2.58, 4.61, 4.54, 3.79, 2.19, 2.42, 4.26 |
|  | Avg (s.e.) | 1.89  (0.17) | 6.29  (0.47) | 1.56  (0.06) | 4.84  (0.33) | 1.26  (0.11) | 6.03  (0.61) | 1.31  (0.09) | 5.67  (0.6) |
| Pre-pupa | 1 | 8.37, 7.88, 5.43, 8.55, 9.33, 9.31, 9.98, 6.98 | 8.04, 9.46, 10.43, 9.78, 10.3, 9.93, 8.56, 7.92, 11.1, 10.1 | 8.43, 8.47, 7.97, 9.05, 8.54, 7.02, 8.08, 6.88, 8.48 | 9.90, 8.85, 8.98, 9.91, 10.9, 8.83 | 7.98, 10.5, 10.2, 7.27, 8.66, 9.39 | 12.8, 13, 10.8, 9.76, 8.62, 8.71, 12.1, 7.43, 8.92 | 8.23, 9.53, 6.39, 7.82, 8.57, 8.75, 8.75, 8.99 | 10.5, 9.45, 9.75, 11.8, 7.13, 11.0, 9.84, 10.9, 10.6 |
|  | 2 | 7.65, 10.0, 10.8, 10.4,  10.7,  8.72, 8.87,  9.28, 10.1,  9.51 | 7.14, 9.84, 8.59, 9.55, 8.93, 7.32 | 6.39, 7.92, 9.31, 7.77, 7.31, 9.60, 8.72 | 9.88, 8.97, 8.64, 8.86, 8.17, 9.33, 8.56, 8.89, 9.15, 10.3 | 7.31, 7.83, 8.14, 8.59, 9.29, 9.29 | 9.34, 10.1, 10.9, 9.56, 8.86 | 8.53, 7.34, 9.39, 7.95, 8.37, 7.56, 8.40, 10.4, 8.0 | 10.3, 8.81, 8.79, 8.83, 9.22 |
|  | Avg  (s.e.) | 8.99  (0.33) | 9.19  (0.29) | 8.12  (0.22) | 9.25  (0.18) | 8.71  (0.29) | 10.1  (0.44) | 8.41  (0.22) | 9.78  (0.32) |

Main effects: GM//NGM type x 2; Tobacco lines x 4; Plants/line/type x 2; Hornworms/plant = 10; Total hornworms = 160.

Two-way ANOVA - 5 Day weight main effects (PROC MIXED): line (P=0.0241); type (P<0.0001). Interactions of main effects: type x line (P=0.1297).

Two-way ANOVA - 10 Day weight main effects (PROC GLM): line (P=0.0873); type (P <0.0001). Interactions of main effects: type x line (P=0.2113).

Two-way ANOVA - Pre-pupa weight main effect (PROC GLM): line (P = 0.1397); type (P<0.0001). Interaction of main effects: type x line (P=0.1397).
