# Supplementary material for: Non-target Effects of Hyperthermostable α-Amylase Transgenic Nicotiana tabacum in the Laboratory and the Field
Source: Front Plant Sci. 2019 Jul 9;10:878. doi: 10.3389/fpls.2019.00878 (PMC6630089; doi:10.3389/fpls.2019.00878)
Supplement: Supplementary file 6 [file Table_5.DOCX]

Table S5. Tobacco hornworm 7 day larval and pre-pupal weight on transgenic and non-transgenic tobacco lines in the field and two-way analysis of variance.

| Weighing date | Plant # | TI95 | | C. Havana | | L. Crittenden | | 81V9 | |
| --- | --- | --- | --- | --- | --- | --- | --- | --- | --- |
|  |  | NGM | GM | NGM | GM | NGM | GM | NGM | GM |
|  |  | Number of days from larvae to each life-stage | | | | | | | |
| Day 7 | 1 | 0.03, 0.06 | 0.15, 0.68, 0.77, 0.29, 0.29, 0.02, 0.42, 0.31 | 0.17, 0.11, 0.07, 0.16, 0.11, 0.04, 0.06 | 0.11, 0.47, 0.12, 0.15, 0.15, 0.35, 0.08, 0.07 | 0.08, 0.21, 0.28, 0.60, 0.11, 0.18 | 0.15, 0.22, 0.24, 0.2, 0.42, 0.27, 0.15, 0.15, 0.32, 0.08 | 0.03, 0.37, 0.21, 0.33, 0.17, 0.17, 0.04, 0.07, 0.09 | 0.07, 0.17, 0.12, 0.51, 0.11, 0.43, 0.26, 0.16, 0.18 |
|  | 2 | 0.26, 0.38 | 0.22, 0.25, 0.08, 0.03, 0.07 | 0.05, 0.03 | 0.11, 0.19, 0.18, 0.28, 0.13, 0.26, 0.21, 0.10 | 0.13, 0.03, 0.01 | 1.37, 0.86, 0.35, 0.32, 0.26, 0.69, 0.56, 0.32 | 0.13, 0.24, 0.18, 0.15, 0.05, 0.14 | 0.43, 1.17, 0.5, 0.58, 0.73, 0.28, 0.25, 0.08 |
|  | Avg  (s.e.) | 0.18  (0.08) | 0.27  (0.06) | 0.09  (0.02) | 0.18  (0.03) | 0.18  (0.06) | 0.4  (0.07) | 0.16  (0.03) | 0.35  (0.07) |
| Pre-pupa | 1 | 9.3 | 7.3, 6.1, 8.4, 8.5, 7.6, 6.8, 7.3 | 6.3, 7.2, 6.5, 8.3, 8.8, 11.8, 10.7 | 7.6, 10.6, 9.3, 7.4, 8.0, 8.1 | 8.2, 7.9, 7.6, 8.3 | 7.3, 7.5, 8.8, 7.2, 6.9, 7.3, 9.0, 7.7 | 8.8, 10.4, 8.5, 8.0, 9.6, 11.1, 8.7, 7.5, 10.8 | 7.3, 10.8, 9.1, 10.1, 8.7, 8.0, 8.3, 8.4 |
|  | 2 | 10.1 | 13.3 | 10.1 | 10.5, 9.5, 9.5 | 10.4 | 9.9, 9.4 | 10.0, 7.7, 8.3, 10.7 | 8.1, 10.0, 12.1 |
|  | Avg (s.e.) | 9.7  (0.4) | 8.1  (0.8) | 8.7  (0.7) | 8.9  (0.4) | 8.0  (0.5) | 8.1  (0.3) | 9.2  (0.3) | 9.2  (0.4) |

Main effects: GM/NGM type x 2; Tobacco lines x 4; Plants/line/type x 2; Trials/line/type x 2; Hornworms/plant = 10; Total hornworms = 160.

Three-way ANOVA - Day 7 weight main effects (PROC GLM): line (P=0.0207); type (P=0.0005); trial (P=0.0443). Interactions of main effects: trial x line (P=0.0403); trial x type (0.0786); line x type (P=0.7322).

Two-way ANOVA Trial 1 - Day 7 weight main effects (PROC MIXED): line (P=0.3427); type (P=0.0127). Interactions of main effects: line x type (P=0.2260).

Two-way ANOVA Trial 2 – Day 7 weight main effects (PROC MIXED): line (P=0.2493); type (P=0.0190). Interactions of main effects: line x type (P=0.0441).

Three-way ANOVA - Pre-pupa weight main effects (PROC GLM): line (P=0.2844); type (P=0.2881); trial (P<0.0001). Interactions of main effects: trial x line (P=0.0498); trial x type (0.0867); line x type (P=0.9879).

Two-way ANOVA Trial 1 - Pre-pupa weight main effects (PROC GLM): line (P=0.0521); type (P=0.0693). Interactions of main effects: line x type (P=0.6805).

Two-way ANOVA Trial 2 - Pre-pupa weight main effects (PROC GLM): line (P=0.3492); type (P=0.3710). Interactions of main effects: line x type (P=0.4650).
